# Epstein-Barr virus as a marker of biological aggressiveness in breast cancer

**DOI:** 10.1038/sj.bjc.6606048

**Published:** 2010-12-21

**Authors:** C Mazouni, F Fina, S Romain, L Ouafik, P Bonnier, J-M Brandone, P-M Martin

**Affiliations:** 1 Laboratoire de transfert d’oncologie biologique, Assistance Publique – Hôpitaux de Marseille, Faculté de Médecine Nord, Marseille, France; 2 Département de chirurgie générale, Institut Gustave Roussy, 114 rue Edouard Vaillant, Villejuif 94805, France; 3 UMR 911 CRO2: Centre de Recherche en Oncologie biologique et Oncopharmacologie, Marseille F-13344, France; 4 Département d’oncologie chirurgicale mammaire et gynécologique, clinique Beauregard, Marseille, France; 5 Départment de chirurgie gynécologique, clinique Bouchard, Marseille, France

**Keywords:** breast cancer, Epstein-Barr virus, HER2, real-time quantitative PCR, thymidine kinase

## Abstract

**Purpose:**

Although a potential role of the Epstein-Barr virus (EBV) in the pathogenesis of breast cancer (BC) has been underlined, results remain conflicting. Particularly, the impact of EBV infection on biological markers of BC has received little investigation.

**Methods:**

In this study, we established the frequency of EBV-infected BC using real-time quantitative PCR (RT–PCR) in 196 BC specimens. Biological and pathological characteristics according to EBV status were evaluated.

**Results:**

EBV DNA was present in 65 of the 196 (33.2%) cases studied. EBV-positive BCs tended to be tumours with a more aggressive phenotype, more frequently oestrogen receptor negative (*P*=0.05) and with high histological grade (*P*=0.01). Overexpression of thymidine kinase activity was higher in EBV-infected BC (*P*=0.007). The presence of EBV was weakly associated with *HER2* gene amplification (*P*=0.08).

**Conclusion:**

Our study provides evidence for EBV-associated BC undergoing distinct carcinogenic processes, with more aggressive features.

A viral aetiology is one recently evocated theory behind the physiopathology of breast cancer (BC) (Glaser *et al*, 2004; de Villiers *et al*, 2005; zur Hausen, 2009). Even though, the mechanistic aspects of cancer induction by infectious agents sound multiples, that is, immunosuppressive, linked to animal–human transmission, direct or indirect oncogenic, there are epidemiological evidences of pathogens involvement in human cancer (zur Hausen, 2009).

Among the putative viruses observed in BC tissue, the presence of the Epstein-Barr virus (EBV), a *γ*-herpes virus, has been reported in a number of studies (Bonnet *et al*, 1999; Fina *et al*, 2001; Glaser *et al*, 2004). The implication of EBV in carcinogenesis associated with other cancers, such as Burkitt's lymphoma, undifferentiated nasopharyngeal carcinoma, as well as Hodgkin's disease, has been well documented (zur Hausen, 1991).

However, the presence and implication of EBV in BC remains controversial. The use of conventional technical approaches (*in situ* hybridisation, immunochemistry and standard PCR) for its detection may explain the conflicting results. Some groups have failed to detect EBV (Chang *et al*, 1992; Gaffey *et al*, 1993; Lespagnard *et al*, 1995; Chu *et al*, 1998; Glaser *et al*, 1998; Dadmanesh *et al*, 2001; Deshpande *et al*, 2002; Herrmann and Niedobitek, 2003; Perrigoue *et al*, 2005), whereas results from others show discrepancy and depended on the methodology used. For instance, although Murray *et al* (2003)could detect EBV nuclear antigen-1 by immunochemistry using 2B4-1 monoclonal antibody, they failed to detect the EBV genome by quantitative PCR. The reasons behind these apparently conflicting results remain to be clarified; however, technical limitations of the assays, dissimilarities in the archival materials and heterogeneity among cluster cells contaminated by the EBV genome may be same. Moreover, EBV positivity has been linked to the presence of latently infected lymphocytes in the tumours (Horiuchi *et al*, 1994; Brink *et al*, 2000) thus, questioning the role of EBV in BC (Chu *et al*, 2001). However, in accordance with other groups (Labrecque *et al*, 1995; Luqmani and Shousha, 1995; Bonnet *et al*, 1999; Chu *et al*, 2001; Huang *et al*, 2003; Preciado *et al*, 2005; Arbach *et al*, 2006; Perkins *et al*, 2006; Tsai *et al*, 2007), we have shown the presence of EBV genetic information in a subset of BC tissue with a specific localisation in the epithelial malignant cells (Fina *et al*, 2001).

Currently, real-time PCR (RT–PCR is increasingly being used for both research and clinical applications. For BC in particular, the detection of *HER2* gene amplification has been validated by comparison with conventional methods, such as FISH (Lamy *et al*, 2006). Analysis using RT–PCR might also help to clearly identify the presence of EBV in BC. However, the use of whole tissue can result in the risk of contamination and this risk has been corrected with the introduction of laser-assisted microdissection (Fina *et al*, 2001). In studies on formalin-fixed sections, micro- and macro-dissected breast tumours have been tested for the presence of multiple regions of the EBV genome with few actually uncovering the viral sequence (McCall *et al*, 2001; Thorne *et al*, 2005). Interestingly, by *in situ* hybridisation using a (35)S-labelled riboprobe for Epstein-Barr encoded RNA 1 and a laser capture microdissection on frozen samples, combined with quantitative PCR (Q-PCR), we showed that EBV localisation was restricted to certain tumour epithelial cell clusters (Fina *et al*, 2001). In accordance with our findings, Arbach *et al* (2006) observed that viral load is variable between tumours and is heterogeneously distributed among morphologically identical tumour cells, some clusters containing high genome numbers compared with others negative for EBV genome within the same specimen.

In the present study, we hypothesised that EBV-infected BC cells might behave differently in comparison to those negative for EBV. In order to test this, we sought to (i) measure the frequency of EBV positivity using RT–PCR and (ii) to compare the biological phenotype of EBV-negative and EBV-positive tumours.

## Materials and methods

### Patients

This study involved 196 primary invasive breast carcinomas, with clinical and pathological characteristics as outlined in Table 1. Patients were consecutively recruited in Marseille France, between May 1996 and December 1998. Tumours were graded according to the Scarff Bloom and Richardson classification (Bloom and Richardson, 1957). Axillary lymph node status was assessed by histological examination. The local Medical Ethics Committee (IRB) approved this laboratory study on stored specimens.

#### Tissue specimens

All tumour samples were histologically examined by a pathologist at the time of initial surgery and stored in liquid nitrogen. Frozen tissue (100 mg) was pulverised with a micro-dismembrator and the frozen powder subsequently used for DNA extraction (Sambrook *et al*, 1982). Cytosols were prepared using a Tris buffer (10 mM Tris-HCl, 1.5 mM EDTA, 10 mM Na2MoO4, 0.5 mM dithiothreitol, 10% glycerol, pH 7.4).

#### Q-PCR analysis

All Q-PCR reactions were performed on an ABI Prism 7700 sequence detection apparatus (Perkin-Elmer Corp., Foster City, CA, USA). The 5′-exonuclease (TaqMan) assay was used. Measurements were performed in duplicates. Levels of *HER2* expression were normalised to those of the somatostatin receptor type II gene *SSTR2* localised on chromosome 17 (q24) and to the glyceraldehyde-3-phosphate dehydrogenase gene *GAPDH* localised on chromosome 12 (p13). Levels of the *Bam*HIC sub-region of the EBV genome were also normalised to *GAPDH*. After normalisation to *GAPDH*, the between-run CVs for *Bam*HIC and *HER*2 internal controls (four series) were less than 10%.

### Q-PCR analysis of *HER2* gene

The Q-PCR reaction conditions used have already been published (Lamy *et al*, 2006). The calibration curve was prepared from normal human genomic DNA (Roche Molecular Biochemicals, Meylan, France). Data were expressed as the HER2/GAPDH and HER2/SSTR2 relative copy number ratio. The human SKBR3 and A431 cell lines, known to, respectively, display HER2 amplification or not, were used as controls.

### Q-PCR analysis of the EBV genome

The Q-PCR analysis of the EBV genome was performed as previously described (Fina *et al*, 2001). Briefly, primers for *Bam*HIC were: sense, 5′-AAA-CAG-GAC-AGC-CGT-TGC-C-3′ (6935–6953); antisense, 5′-AAG-CCT-CTC-TTC-TCC-TTC-CCC-3′ (7036–7016) and the probe was 5′-FAM-TTT-CGG-ACA-CAC-CGC-CAA-CGC-T-TAMRA-3′ (6961–6983). The cycling conditions for both *Bam*HICs were as follows: 95°C for 15 min; 45 cycles of 94°C for 20 s, and 55°C for 20 s. Amplification was performed in a 50-*μ*l reaction volume with a buffer consisting of 10 mmol l^–1^ Tris-HCl (pH 8.3; 25°C), 50 mmol l^–1^ KCl, 10 mmol l^–1^ ethylenediamine tetraacetate and 5 mmol l^–1^ MgCl2 in the presence of 200 *μ*mol l^–1^ deoxy(d)-ATP, dCTP and dGTP, 400 *μ*mol l^–1^ dUTP, 200 nmol l^–1^ of each primer, 200 nmol l^–1^ probe, 1 U Amp Erase UNG (Perkin-Elmer Corp.), and 1.25 U AmpliTaq Gold polymerase (Perkin-Elmer Corp.). To quantify the EBV genome load in the tissues, genomic DNA prepared from the Raji cell line, containing 50 integrated EBV copies per cell was used. Serial dilutions of DNA were prepared from 1 ng to 0.1 pg equivalent to 15000–1.5 copies of EBV genome, respectively. Absolute quantification of the *Bam*HIC standard curve involved comparison against normal human genomic DNA (Roche Molecular Biochemicals). The calibration curve for GAPDH was directly prepared from normal human genomic DNA. *Bam*HIC data were expressed as the number of *Bam*HIC copies per 100 ng *GAPDH*. Normal human genomic DNA and controls lacking DNA always remained negative in the *Bam*HIC Q-PCR analyses.

#### Biochemical assays

Oestrogen receptors and progesterone receptors (PRs) (EIA, Abbott Laboratories, Chicago, IL, USA), as well as urokinase plasminogen activator (UPA) and plasminogen activator inhibitor type 1 (PAI-1) (UPA Imubind no 894 and PAI-1 Immubind no 821, both from American Diagnostica, Greenwich, CT, USA) were measured with enzyme immunoassays. Thymidine kinase (TK) activity was determined by a radioenzymatic phosphorylation assay (TK-REA, Sangtec Medical, Bromma, Sweden) optimised to detect the fetal TK1 isoenzyme, as previously described (Romain *et al*, 1994). Quality control was assured by frequent testing with internal controls. The EORTC standards were also used for oestrogen receptor and PR (Geurts-Moespot *et al*, 2000).

#### Statistical analysis

Associations between categorical variables were tested by the *χ*^2^-test. Relationships between categorical and continuous variables were examined using the Mann–Whitney test, or in the case of more than two ordered categories, by the Kruskal-Wallis test. A *P*-value of <0.05 was considered significant. The *HER2* gene was considered amplified when the *HER2/GAPDH* relative copy number was ⩾2.0. A threshold value of 4.0 was used to define a strong *HER2* amplification. Samples with receptor content ⩾20 fmol mg^–1^ protein were classified as oestrogen receptor or PR positive. Cut-offs corresponding to the seventy-fifth percentiles in the distributions were used to dichotomise UPA, PAI-1 (Bouchet *et al*, 1999) and TK (Romain *et al*, 1995, 2000, 2001) as previously recommended.

## Results

### Patient characteristics

The demographics of the studied patients are reported in Table 1. The tumours had a diameter of more than 2 cm in 97 patients (49.5%). In all, 76 patients (38.8%) had positive axillary lymph nodes. The histological differentiation was determined as grade 3 in 56 tumours (28.6%).

*HER2* gene amplification was detected in 15.3% (*n*=30) of the analysed BCs, with the *HER2/GAPDH* ratio for amplified cases ranging from 2.0–22.1 (median 4.5). Among the *HER2*-amplified tumours, 43.3% (*n*=13) showed moderate *HER2* amplification (*HER2/GAPDH* ratio 2.0–4.0) and 56.7% (*n*=17) a strong amplification (*HER2/GAPDH* ratio⩾4.0).

### Q-PCR analysis of the EBV genome

To ensure that the presence of EBV was related to epithelial cells, as previously described (Fina *et al*, 2001). Tissue sections were microdissected with a PixCell II LCM system (Arcturus Engineering, Mountain View, CA, USA). For each tumour analysed, several epithelial areas (approximatively 5 × 10^3^ cells) were independently captured; stromal areas without infiltrating malignant epithelial cells were pooled to provide a sufficient number of GAPDH copies. Cell populations were estimated to be homogeneous as determined by microscopic visualisation. DNA from laser-captured cells was extracted and subsequently used for Q-PCR.

The presence of the *Bam*HIC sub-region of the EBV genome was used to define EBV positivity. EBV was detected in 65 (33.2%) of the 196 investigated BCs. Among the positive tumours, the load of EBV genome varied from 0.08 to 810.8 *Bam*HIC copies per 100 ng *GAPDH* (median 1.4). Fibrocystic diseases (*n*=3), fibroadenomas (*n*=6), phyllode tumours (*n*=4) and normal mammary tissue obtained from mammoplasty (*n*=2) were also analysed. They were all found to be negative, with the exception of one phyllode tumour (0.43 *Bam*HIC copies per 100 ng *GAPDH*).

### Detection of the EBV genome and patient characteristics

Table 1 shows the frequency of the EBV genome according to the characteristics of the patient and tumour.

Tumour positive for EBV presented markers of proliferation. Thus, the proportion of EBV-positive samples was significantly higher among the high-grade tumours (16.2% for grade I, 32.0% for grade II and 46.4% for grade III, *P*=0.01). EBV-positive samples were more frequent among those of ER-negative (45.4%) compared with ER-positive tumours (29.6%) (*P*=0.05). Among the tumours with high TK, 49.0% displayed the EBV genome compared with 27.9% of those with low TK (*P*=0.007). In contrast, no significant link was observed between the detection of the EBV genome and age at diagnosis, tumour size, lymph node involvement, PR, UPA or PAI-1 status.

To quantitatively assess the relation between the EBV presence and pathological markers, we have studied the load of EBV genome. We confirmed that *Bam*HIC copy numbers were higher among high-grade tumours (*P*=0.006) and between those ER-negative (*P*=0.01) and high TK value (*P*=0.009). Other relationships were not significant (Table 2).

### Detection of the EBV genome and amplification of *HER2*

A weak association was observed between EBV genome presence and *HER2* amplification. Subgroups with EBV– HER2– (*n*=115) and EBV+ HER2+ (*n*=14) status represented 65.8% of the investigated patients (*P*=0.09). When using the Mann–Whitney test, EBV-positive tumours showed the highest *HER2* copy numbers though the difference did not reach significance (*P*=0.08).

## Discussion

The aim of this study was to investigate the presence of EBV in BC, alongside possible associations with clinicopathological factors and biological tumour features that either mark the natural history of the disease or determine the therapeutic outcome. The analysed biological factors were selected on the basis of their high utility score in the tumour marker grading system (Isaacs *et al*, 2001), with evidence coming either from prospective trials or meta-analysis (ER, PR, HER2, UPA and PAI-1), or at least from large retrospective studies (TK).

The implication of EBV in the aetiology of BC has been addressed in other series, including a multicentric study carried out by our group (Fina *et al*, 2001). In accordance with tour analysis, the presence of EBV was showed to be restricted in the epithelial cells (Fawzy *et al*, 2008; Trabelsi *et al*, 2008; Joshi *et al*, 2009). EBV has been evocated along with other viruses, such as the papillomavirus (de Villiers *et al*, 2005; Kulka *et al*, 2008) or polyomavirus (Berebbi *et al*, 1990), as well as cytomegalovirus (Richardson *et al*, 2004). One of the controversies surrounding EBV as a causal agent in BC has been its potential coincidental presence as no virus-related physiopathological effects have emerged from pathological observations. However, one interesting epidemiological study provided some arguments in favour of a role and a potential explanation relating to the stage of mammary gland development. Indeed Yasui *et al* (2001) showed a correlation between the incidence of infectious mononucleosis and the risk of BC. Particularly, an increase in age corresponding to a later stage of mammary gland development at infectious mononucleosis onset seemed to increase the risk for BC. We have also observed this potential link between the incidence of BC and hormonal status in one of our previous studies with the polyomavirus (Berebbi *et al*, 1990). Our evaluation of oncogenicity in nude mice showed that mammary tumour induction was oestrogen-dependent during a short period after polyomavirus injection. This sensitivity of the mammary gland to virus exposure corresponds to an oestradiol-mediated modification of the target organ occurring during ductal development (Berebbi *et al*, 1990). After this developmental period, the mitogenic stimulus induced by hormones is no longer necessary. The influence of hormonal environment during the critical period of mammary gland development thus determines the future carcinogenesis process and the pool of hormone-responsive epithelial cells (Nandi *et al*, 1995). However, the analysis of changes in EBV immunoglobulins (Ig) showed discrepant results; thus, although Cox *et al* (2010) failed to show an association with the risk of BC in Ig taken before and after the development of BC, in contrast Joshi *et al* (2009) observed no difference that mean anti-EBNA-1 IgG levels were significantly higher in BC patients as compared with benign breast disease.

The Q-PCR method has been used here to investigate EBV in a large series of BCs. Overall, 33.2% of the 196 frozen tumours analysed contained the *Bam*HIC sub-region of the EBV genome that encodes the Epstein-Barr encoded RNAs. In our previous multi-centre study (Fina *et al*, 2001), the samples from our centre showed a positive ratio of 26.7% by standard PCR. The higher percentage of EBV-positive tumours observed in the present study may be related to the size of the tumours samples analysed, (100 mg), and to the sensitivity of Q-PCR. Two other investigators have also found EBV by PCR in frozen tissues by PCR (Labrecque *et al*, 1995; Bonnet *et al*, 1999). The absence of detection (Gaffey *et al*, 1993; Lespagnard *et al*, 1995) or the low detection rate even by RT–PCR (McCall *et al*, 2001) that has elsewhere been reported elsewhere could be due to result from the use of fixed tissues. Indeed, it has been demonstrated that fixation generates both PCR-inhibitory components (Satoh *et al*, 1998; Kösel *et al*, 2001; kalkan *et al*, 2005) and sequence alterations (Williams *et al*, 1999; Amarante and Watanabe, 2009). Inhibition of the viral DNA PCR amplification was most likely the case for the study of McCall and colleagues (McCall *et al*, 2001). Kalkan *et al* (2005) and Thorne *et al* (2005) detected EBV genome in epithelial and also in normal cells. In these studies, low amounts of template DNA was probably used as the tissues were microdissected and DNA amplificability was controlled by HER2 detection. In our BC samples, the loads of EBV genetic information (*Bam*HIC per 100 ng DNA) ranged far below the range of HER2 values (*HER2*/*GAPDH* copy number). The high heterogeneity in EBV detection that has been shown within individual tumours also needs to be considered (Fina *et al*, 2001).

In this study, we observed a difference in clinical and biological profiles between EBV-positive and EBV-negative cancers. In accordance with Tsai *et al* (2007) though in contrast to Murray *et al* (2003), we found no correlation between the presence of EBV and nodal status. Here, we have investigated markers of epithelial cells (ER, PR, grade and TK), whereas UPA and PAI-1, which are markers of stromal-epithelial interactions, associated with tumoural invasion process. In line with this result, no association was observed with the biological factors (UPA or PAI-1) related to tumour invasion. However, we did confirm that the proportion of EBV-positive samples is higher among the high-grade and the ER-negative cancers (Bonnet *et al*, 1999; Murray *et al*, 2003). These latter biological factors related to differentiation status (Rose *et al*, 1985; McGuire *et al*, 1986; Murray *et al*, 2003) were strongly associated with EBNA-1 as detected by immunostaining. Altogether these results confirm the epithelial presence of EBV as only correlations with epithelial markers were observed and not with the markers of stromal compartment. Interestingly, we showed a positive association between the presence of EBV and high cytosolic TK enzyme activity. High expression of this enzyme involved in the DNA synthesis salvage pathway has previously been associated with large tumour size, high histological grade and steroid hormone receptor negativity (Romain *et al*, 2000). The TK encoded by the EBV is localised in the centrosome, a localisation observed in diverse cell types whether the protein is expressed independently or in the context of lytic EBV infection (Gill *et al*, 2007). Although EBV TK is an early gene, it was nevertheless found to be transcribed with a significant delay compared with other early-class RNAs (Yuan *et al*, 2006). The commercial assay used to access TK activity has been optimised for the TK1 isoenzyme. The link between EBV and TK supports the notion that EBV is associated with fast-growing tumours. It agrees with data suggesting that DNA tumour viruses suppress the transcriptional downregulation of TK activity during the eukaryotic cell cycle (Hengstschläger *et al*, 1994), and that nasopharyngeal carcinomas with detectable EBV LMP1 protein grow faster than the non-expressing ones (Hu *et al*, 1995). Concerning the link between EBV positive tumours and high TK expression, we sequenced mRNA and found that the expression of *TK* gene found in EBV-positive tumours is of human origin, not viral (data not shown). This human TK differs from the one deposited by Bradshaw and Deininger in the Genbank database (Gilles *et al*, 2003). In a previous study, Huang *et al* (2003), detected the EBV-encoded lytic transactivator protein ZTA in 7 out of 10 BCs. Interestingly, ZTA specifically binds the CCAAT motif (C/EBP*α*, enhancer binding protein *α*) of the TK1 human gene promoter, which suggests a functional role in the activation of TK1 transcription.

In this study, the presence of EBV was only weakly associated with *HER2* amplification. This result, together with the fact that EBV and HER2 correlate differently with other tumour features, suggests that the viral infection and the gene amplification occur at different times during BC progression. In BCs, both EBV (Bonnet *et al*, 1999) and HER2 (Révillion *et al*, 1998; Yamauchi *et al*, 2001) have been associated with a lack of oestrogen receptors.

In conclusion, we confirmed the presence of EBV in one third of BC. Moreover, EBV-positive tumours presented with a more aggressive phenotype that could be useful when considering potential therapeutic targets. In particular, the high TK levels could confer resistance to chemotherapy.

## Figures and Tables

**Table 1:** Frequency of EBV positivity according to patient and tumour characteristics

**Characteristics**	***n***	**EBV+ (%) (*n*=65)**	**EBV− (%) (*n*=131)**	***P*-value**
All	196	33.2	66.8	
				
*Age (years)*				
<50	54	17 (31.5)	37 (68.5)	NS
⩾50	142	34 (33.8)	63 (66.2)	
				
*Tumour size (pT)*				
<2 cm	99	31 (31.3)	68 (68.7)	NS
>2 cm	97	34 (35.0)	63 (65.0)	
				
*Nodal status (pN)*				
N−	120	30.0	70.0	NS
N+	76	38.2	61.8	
				
*Histological grade*				
I	37	6 (16.2)	31 (83.8)	0.01
II	103	33 (32.0)	70 (68.0)	
III	56	26 (46.4)	30 (53.6)	
				
*ER*				
Negative	44	20 (45.4)	107 (54.6)	0.05
Positive	152	45 (29.6)	45 (70.4)	
				
*PR*				
Negative	62	23 (37.1)	39 (62.9)	NS
Positive	134	42 (31.3)	92 (68.7)	
				
*UPA*				
Low	147	14 (34.7)	35 (65.3)	NS
High	49	51 (28.6)	96 (71.4)	
				
*PAI-1*				
Low	147	48 (32.6)	99 (67.4)	NS
High	49	17 (34.7)	32 (65.3)	
				
*TK*				
Low	147	24 (27.9)	25 (72.1)	0.007
High	49	41 (49.0)	106 (51.0)	
HER2 amplification		14	16	0.13

Abbreviations: % −, % +=percentage of tumours negative and positive for EBV, respectively; EBV=Epstein-Barr virus; ER=oestrogen receptor; NS=not significant; PAI-1=plasminogen activator inhibitor 1; PR=progesterone receptor; TK=thymidine kinase; UPA=urokinase plasminogen activator.

**Table 2:** Mean EBV load (*Bam*HIC copies) according to biological characteristics

**Characteristics**	**Mean EBV load (*Bam*HIC copies per 100 ng *GAPDH*)**	***P*-value**
*Age (years)*		
<50	0.79	0.42
⩾50	7.84	
		
*Tumour size (pT)*		
<2 cm	1.28	0.70
⩾2 cm	10.60	
		
*Nodal status (pN)*		
N−	1.40	0.34
N+	13	
		
*Histological grade*		
I	0.32	0.006
II	2.58	
III	15.7	
		
*ER*		
Negative	1.64	0.01
Positive	20.58	
		
*PR*		
Negative	1.83	0.20
Positive	14.67	
		
*UPA*		
Low	7.48	0.56
High	1.12	
		
*PAI-1*		
Low	7.21	0.75
High	1.93	
		
*TK*		
Low	1.87	0.009
High	17.95	
No HER2 amplification	27.9	0.10
HER2 amplification	1.9	

Abbreviations: ER=oestrogen receptor; PAI-1=plasminogen activator inhibitor 1; PR=progesterone receptor; TK=thymidine kinase; UPA=urokinase plasminogen activator.
